# Stabilization Splint Therapy for Patients with Temporomandibular Disorders Improves Opening Movements and Jaw Limitation and Attenuates Pain by Influencing the Levels of IL-7, IL-8, and IL-13 in the Gingival Crevicular Fluid

**DOI:** 10.3390/medicina61030375

**Published:** 2025-02-21

**Authors:** Renata Sikora, Kristina Duspara, Anita Matić, Ana Petrović, Kristina Kralik, Robert Smolić, Miroslav Sikora, Martina Čalušić Šarac, Kristina Bojanić, Martina Smolić

**Affiliations:** 1 Faculty of Dental Medicine and Health Osijek, J. J. Strossmayer University of Osijek, 31000 Osijek, Croatia; rsikora@fdmz.hr (R.S.); dusparakristina5@gmail.com (K.D.); anitamatic@fdmz.hr (A.M.); anapetrovic@fdmz.hr (A.P.); rsmolic@fdmz.hr (R.S.); msikora@fdmz.hr (M.S.); mcsarac@fdmz.hr (M.Č.Š.); kbojanic@fdmz.hr (K.B.); 2 Health Center Osijek-Baranja County, 31000 Osijek, Croatia; 3 Faculty of Medicine Osijek, J. J. Strossmayer University of Osijek, 31000 Osijek, Croatia; kristina.kralik@mefos.hr; 4 Public Health Scientific Institution Medical Center “Dr. Mustafa Sehovic”, 75000 Tuzla, Bosnia and Herzegovina

**Keywords:** biomarkers, gingival crevicular fluid, interleukin-7, interleukin-8, interleukin-13, stabilization splint

## Abstract

*Background and Objectives:* In recent years, numerous studies have investigated and analyzed the levels of molecular biomarkers of temporomandibular disorders (TMD) from various tissue samples and body fluids. However, no study has investigated gingival crevicular fluid (GCF) in TMD patients. The purpose of this study was to determine the concentrations of pro-inflammatory cytokines in GCF before and after stabilization splint (SS) therapy in patients with painful TMD, to investigate whether SS administration causes changes in the concentrations of pro-inflammatory cytokines. An additional aim was to investigate the relationship of GCF cytokine levels with chronic pain intensity and clinical parameters. *Materials and Methods:* This prospective cohort study included 36 patients who were diagnosed with painful TMD using the Diagnostic Criteria for TMD (DC/TMD). GCF samples were collected at baseline before SS treatment (T0) and at one month (T1) and three months (T2) after the start of therapy. Customized ProcartaPlex Multiplex assays from eBioscience (Invitrogen™, Thermo Fisher Scientific, Viena, Austria) were used for the quantitative analysis of pro-inflammatory cytokines (IL-1β, IL-6, IL-7, IL-8, IL-13, and TNF-α). Patients filled out Croatian versions of questionnaires for self-assessment from Axis II DK/TMP: Graded Chronic Pain Scale (v2) (GCPSv2) and Jaw Function Limitation Scale-20 (JFLS-20). *Results:* The results showed that the GCF levels of IL-7 (Friedman’s test, *p* = 0.008) and IL-13 (Friedman’s test, *p* = 0.003) were significantly decreased at T2. The GCF level of IL-13 was in negative correlation with chronic pain grade score at T2 (Rho = −0.333), while the GCF level of IL-8 was in positive correlation with mobility limitation (Rho = 0.382) at T1. *Conclusions:* The results indicate that SS therapy might have a role in reducing inflammation and that the GCF could be a valuable medium for assessing molecular biomarkers.

## 1. Introduction

Temporomandibular disorder (TMD) refers to painful and non-painful conditions that affect the masticatory muscles, temporomandibular joints (TMJ), and surrounding tissues [1]. With a frequency of 34% worldwide and varying rates by continent (South America—47%; Asia—33%; North America—26%; and Europe—29%), TMDs are a significant global health concern. Women have a higher prevalence, ranging from 9% to 56% on average, and the highest incidence, 41%, occurs in those aged 18 to 60 [2]. The main symptoms are persistent pain, limitations of jaw function, and sounds (clicking, grinding) in the TMJ area [1,3]. Treatment consists of various therapeutic methods such as pharmacotherapy, physiotherapy, therapy with various occlusal appliances, surgical treatment, cognitive-behavioral, educational, and self-control therapy [4]. Current scientific findings point to the advantage of conservative and non-invasive treatments [4,5]. The most commonly used method is stabilization splint therapy (SS) [6,7]. SS’s therapeutic effect is attributed to a variety of factors, including changing the position of the condyle in the articular fossa, stabilizing occlusion, and reducing neuromuscular activity [6]. However, due to the conflicting results of different studies, the exact mechanism of action of the stabilization splint on the relief of symptoms in TMD patients cannot be explained with certainty [6,8]. 

The etiopathogenesis is multifactorial and complex [2,3,5], making it difficult for clinicians to make an accurate diagnosis and choose the best treatment. Therefore, new insights into the pathophysiology of TMD as well as diagnosis, prognosis, and treatment are needed.

An inflammatory response is considered one of the possible underlying mechanisms of painful TMD which has drawn the attention of researchers to study pro-inflammatory cytokines as pain biomarkers [9,10,11]. The pain occurs due to mechanical stimulation of peripheral nociceptors leading to hypoxia and increased levels of inflammatory mediators such as cytokines, chemokines, and neuropeptides [10,11,12]. Moreover, studies have reported an association between pro-inflammatory cytokines and pain in TMD patients compared to healthy control groups and that cytokines play an important role in the onset and progression of the disease [11,12]. TMD patients have higher levels of tumor necrosis factor-alpha (TNF-α), interleukin 1 beta (IL-1β), and interleukin 6 (IL-6) in the synovial fluid of their TMJs compared to healthy controls, which is also linked to increased pain levels [11,12,13,14,15]. In addition, elevated levels of IL-6, interleukin 7 (IL-7), interleukin 8 (IL-8), and interleukin 13 (IL-13) were found in the masseteric muscle of patients with TMD [16]. 

The level of molecular biomarkers is determined from tissue samples and body fluids such as blood serum and plasma, saliva, synovial fluid, and cerebrospinal fluid [11,12]. An ideal biomarker should be widely available, inexpensive, and reliable, with high sensitivity and specificity for detecting and monitoring disease or predicting treatment outcomes [17]. In the search for biomarkers, numerous studies have been conducted on serum/plasma and synovial fluid [11,12,14]; however, more recent studies show increasing interest and the need for less invasive and approachable samples such as saliva and gingival crevicular fluid (GFC). The GCF is a serum transudate secreted from the gingival plexus of blood vessels in the healthy gingival sulcus which is localized between the tooth and the unattached gingiva [18,19]. During inflammation, serum transudate converts into an inflammatory exudate fluid, rich in serum proteins, diverse types of cells, inflammatory mediators, electrolytes, enzymes, etc. [18,19]. A recent study reported that the cytokine profile in the GCF sample showed less variation between healthy individuals compared to saliva, making GFC a more reliable source for biomarker search [20]. An increasing number of studies demonstrate that GCF is already recognized as a valuable source of biomarkers for various oral and systemic diseases and that analytes from GCF can successfully reflect local and systemic inflammation. For instance, numerous studies have investigated GCF in periodontitis, peri-implantitis, cardiovascular diseases, inflammatory bowel diseases, diabetes mellitus, Alzheimer’s disease, chronic hepatitis C, etc. [21,22,23,24,25,26,27]. However, we have not found any study investigating GCF in patients with TMD. 

We hypothesized that stabilization splint therapy in patients with temporomandibular disorders leads to a decrease in the level of pro-inflammatory cytokines. Therefore, the aim of this study was to determine the concentrations of pro-inflammatory cytokines in the GCF before and after SS therapy in patients with painful TMD in order to observe if SS administration affects pro-inflammatory cytokine levels. An additional aim was to investigate the association of GCF levels of cytokines with chronic pain intensity and clinical parameters.

## 2. Materials and Methods

### 2.1. Study Design and Ethical Concerns 

This was a prospective cohort study at three time points: baseline before stabilization splint (SS) therapy (T0), at one-month follow-up (T1), and at three-month follow-up after the treatment start (T2). The research was approved by the Review Boards of Health Center Osijek (registration number: 03-2673-1/22, date of approval: 24 November 2022) and the Faculty of Medicine Osijek, Josip Juraj Strossmayer University of Osijek (registration number: 2158-61-46-24-123, date of approval: 24 May 2023) and was carried out under the ethical principles of the Declaration of Helsinki. All patients who participated in the study were given written and verbal information about it and were required to sign a consent form. The study protocol was registered at the International Clinical Trials Register (ClinicalTrials.gov ID: NCT06043024, date of registration: 15 September 2023). 

### 2.2. Participants

The study included thirty-six patients (median age 35) referred to the Specialist Prosthodontic Dental Clinic, Health Center of Osijek-Baranja County, Osijek, Croatia with pain symptoms in the orofacial region between 16 August 2023 and 16 August 2024 (Table 1). Inclusion criteria were myalgia, arthralgia, TMD-related headaches, and painful disk displacement (with or without reduction) lasting at least three months with an average pain intensity of ≥3/10 on a numeric rating scale (NRS). All diagnoses were made based on Axis I of the Diagnostic Criteria for Temporomandibular Disorders (DC/TMD) [28]. Exclusion criteria included degenerative joint disease, temporomandibular joint (TMJ) subluxation, a history of head trauma, orofacial pain unrelated to temporomandibular disorders, periodontitis, and pregnancy. Patients with acute infections, chronic diseases, autoimmune diseases, those who had taken anti-inflammatory drugs and muscle relaxants 48 h prior to data collection, and those taking immune-system-affecting drugs (such as antiproliferative immunosuppressants, corticosteroids, disease-modifying antirheumatic drugs (DMARDs), anti-lymphocyte monoclonal antibodies, antidepressants, and antiepileptics) were all excluded. The study did not include patients with prostheses and those already undergoing orthodontic treatment or occlusal splint therapy. 

The clinical examination, crevicular fluid sample collection, splint delivery, and adjustments were performed by the first author (R.S.), an experienced clinician and specialist in dental prosthetics.

### 2.3. Clinical Examination and Questionnaires

A detailed medical and dental history was recorded during the first visit, and a clinical examination was performed at the baseline (T0) and follow-ups (T1 and T2). One or more diagnoses were established for each participant using patient assessment instruments according to Axis I of DC/TMD [29]. The DC/TMD Symptom Questionnaire is used for a more detailed assessment of jaw pain, temporal region headache, and their modifying factors, which is also part of Axis I DC/TMD. Clinical measurement consisted of pain-free mouth opening, maximum unassisted opening, maximum assisted opening, and lateral and protrusive movements measured between the maxillary and mandibular reference teeth with the ruler in millimeters (mm). All clinical measurements were performed according to the clinical examination protocol of DC/TMD [29]. Patients filled out Croatian versions of questionnaires for self-assessment from Axis II DK/TMP: Graded Chronic Pain Scale (v2) (GCPSv2) and Jaw Function Limitation Scale-20 (JFLS-20) [29]. Pain intensity and pain-related disability were assessed using the Graded Chronic Pain Scale (v2) (GCPSv2) questionnaire [30,31]. The GCPSv2 questionnaire consists of 8 items: 3 for pain intensity, 4 for function, and 1 for the number of painful days. Items 2–4 and 6–8 consist of a scale with values ranging from 0 (no pain/no interference) to 10 (pain as bad as could be/unable to carry on any activities). The grade of chronic pain is calculated by combining the characteristic pain intensity score and the disability score which enables us to classify patients into five categories, from 0 (no pain) to 4 (severe limitations). The jaw function limitation was assessed by a JFLS-20 questionnaire consisting of 20 questions with answers on a Likert scale ranging from 0 (no limitation) to 10 (severe limitation). These twenty questions are divided into three categories: limitations in mastication, mobility, and verbal and emotional expression. Questionnaires are validated and translated into the Croatian language [29]. Participants were asked to complete questionnaires before beginning SS treatment (T0) and at follow-up visits (T1, T2).

### 2.4. Stabilization Splint Fabrication

Each participant received a custom-made stabilization splint (SS) for the upper jaw. Upper and lower jaw impressions (Alginate Plus fast set, Henry Schein, Monzastrasse 2a, D-63225 Langen, Germany) were taken, and cast models were made in type IV hard plaster (GC FUJIROCKtm EP, GC EUROPE N.V., B-3001 Leuven, Belgium). After that, the cast models were placed into the Artex^®^ articulator (Amann Girrbach, Koblach, Austria) using a facebow (Artex, Amann Girrbach, Koblach, Austria) and occlusal records of centric relation, left and right laterotrusion, and protrusion. The SS was made of hard acrylate resin ORTOpoli (Polident d.o.o., Volčja Draga 42, SI-5293 Volčja Draga, Slovenia) about 1.5 mm thickness at the posterior teeth area, covering all the upper teeth and making contacts with opposite teeth during the maximal intercuspation. In addition, the SSs were designed with an incisal ramp for anterior and canine guidance during the protrusion and laterotrusion movements, thus eliminating any occlusal interference. All SSs were made by one dental technician in the same dental laboratory. Patients were instructed to wear the occlusal splints during the night and how to perform SS daily hygiene routine.

### 2.5. Gingival Crevicular Fluid (GCF) Sampling

The GCF samples were collected on the day the SS was administered to the patient (T0) and at follow-up examinations (T1 and T2) between 8 AM and 12 AM due to potential diurnal variation in some analytes. The collection procedure was carried out as previously described [32,33,34]. The participants were asked to wash their teeth at least one hour before their appointment at our clinic to avoid sample contamination with plaque and to reduce potential gingival inflammation. Any remaining plaque was gently removed with a rotating dental brush and a 5-min wait was observed before starting the sample collection. The oral cavity was isolated with cheek retractors and cotton rolls around the sample site area. Saliva from the sample site was washed with water and gently dried for 5 s with air from the syringe. A GCF sample was collected with sterile tweezers and no. 20 standardized sterile paper points (Cerkamed, ul. Kwiatkowskiego 1, 37-450, Stalowa Wola, Poland). Four paper points were applied into distobuccal gingival crevices of the maxillary incisors until a slight resistance was felt and held for 30 s each to absorb the GCF. After that, they were placed in a coded, precooled Eppendorf test tube with 250 µL of 0.9% NaCl solution and stored at +4 °C. On the same day, the samples were centrifuged at 3000 rcf/15 min at +4 °C, paper points were removed, and the supernatant was stored at −80 °C until laboratory analysis. 

### 2.6. Laboratory Analysis

Customized ProcartaPlex Multiplex Immunoassays from eBioscience (Invitrogen™, Thermo Fisher Scientific, Bender MedSystems GmbH Campus Vienna Biocenter 2 A-1030 Vienna, Austria) were used for quantitative analysis of pro-inflammatory cytokines (interleukin 1 beta (IL-1β), interleukin 6 (IL-6), interleukin 7 (IL-7), interleukin 8 (IL-8), interleukin 13 (IL-13), tumor necrosis factor-alpha (TNF-α)). The principle is based on beads for protein detection and quantification according to the use of Luminex^®^ xMAP^®^ (multi-analyte profiling) (Bio-Techne GmbH Borsigstrasse 7A, 65205 Wiesbaden, Germany). The entire protocol was carried out according to the manufacturer’s instructions (Affymetrix Inc., Vienna, Austria). The plate was read in the Luminex^®^ xMAP^®^ device (Bio-Techne GmbH, Wiesbaden, Germany). The levels of the tested analytes were determined using ProcartaPlex Analyst v 1.0. eBioscience, Affymetrix, Vienna, Austria).

### 2.7. Statistical Analysis

To calculate the sample size, the statistical method of calculating the appropriate number of participants was applied as follows; to observe a mean effect (f = 0.25) in the difference in continuous variables at three measurement points, with a significance level of 0.05 and a power of 0.80, the minimum required sample size is 28 participants (G*Power version 3.1.2) [35]. 

Categorical data were presented in terms of absolute and relative frequencies. The McNemar–Bowker or Marginal Homogeneity Tests were used to examine differences in categorical variables between measurements. The Shapiro–Wilk test was used to determine the normality of the numerical variable distributions. The median and interquartile ranges were used to describe numerical data. Differences in continuous variables between measurements were analyzed using the Friedman test (post hoc Conover test). Spearman’s rank correlation coefficient (ρ) was used to analyze the relationship between continuous variables. All *p*-values were two-tailed, and the significance level was set at α = 0.05. MedCalc^®^ Statistical Software version 22.006 (MedCalc Software Ltd., Ostend, Belgium; https://www.medcalc.org; 2024) was used to analyze the data.

## 3. Results

### 3.1. Characteristics of Participants

**Figure 1:**
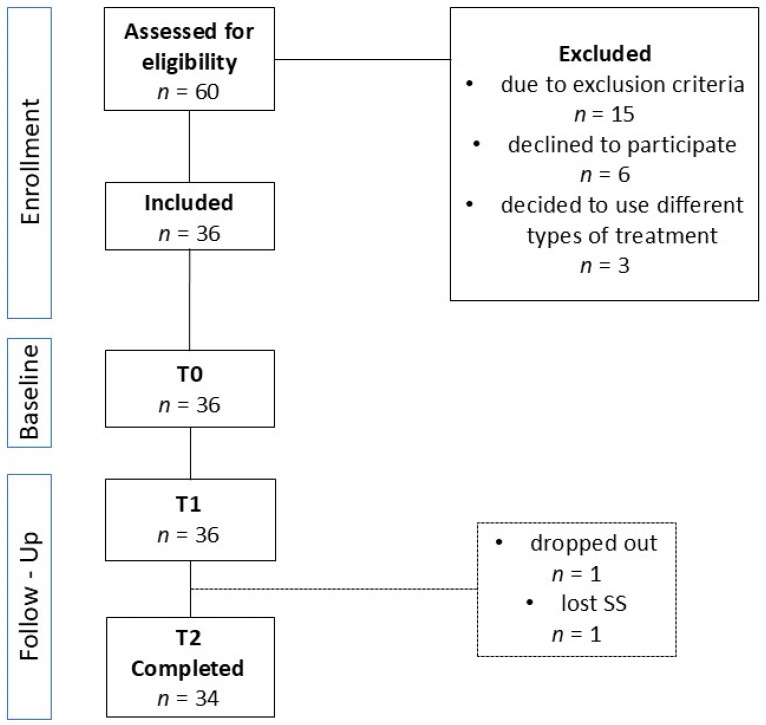
Flowchart of participants.

Sixty patients were considered for inclusion in the study, of which twenty-four patients were excluded for various reasons, as shown in the flowchart (Figure 1). Finally, the research was conducted on 36 subjects, of which 5 (14%) were men and 31 (86%) were women. Table 1 shows the sociodemographic and pain characteristics of participants. The median age of the participants is 35 years, ranging from a minimum of 19 to a maximum of 64 years. Sixteen (44%) participants were married. Regarding level of education, 12 (33%) respondents had completed high school or had finished college. Participants reported a median pain duration of one year, ranging from 5 months to a maximum of 3 years. The median current pain intensity on a 0–10 numeric rating scale (NRS) is 4, while the median average pain intensity is 5. 

### 3.2. Clinical Outcomes

Regarding clinical measurements of opening movements, pain-free mouth opening was significantly higher 3 months after the start of therapy (Friedman’s test, *p* < 0.001), while maximum unassisted and assisted opening was significantly less before splint therapy compared to the other two measurements (Friedman’s test, *p* < 0.001) (Table 2). Right lateral movement is significantly different in all three measurements (Friedman’s test, *p* < 0.001) (Table 2). In the case of left lateral movement it is significantly lower before splint therapy compared to 1 month after the start of treatment (Friedman’s test, *p* = 0.003), while protrusion is significantly smaller before splint therapy compared to the other two measurements (Friedman test, *p* = 0.02) (Table 2).

Chronic pain was assessed with the GCPSv2 questionnaire at all three time points. Before SS therapy, 13 (36%) participants had moderate or severe limitations due to pain and three months after the start of the treatment there was a significantly higher number of participants without pain and with low-intensity pain without disability (Test of marginal homogeneity, *p* < 0.001, Chramer’s V = 0.334) (Table 3).

Mastication, mobility, and verbal and emotional expression limitation three months after the start of therapy were significantly lower compared to other measurements. The overall scale is significantly different for all measurements (Friedman’s test, *p* < 0.001) (Table 4).

### 3.3. Concentrations of Pro-Inflammatory Cytokines in GCF and Correlation with Clinical Outcomes

The concentration of pro-inflammatory cytokines IL-1ß, IL-6, IL-7, IL-8, IL-13, and TNFα in gingival crevicular fluid (GCF) was examined. We observed that GCF levels of IL-7 (Friedman’s test, *p* = 0.008) and IL-13 (Friedman’s test, *p* = 0.003) were significantly lower at three-month follow-up compared to baseline and one-month follow-up (Table 5).

Using Spearman’s correlation coefficient, the association of GCF levels of pro-inflammatory cytokines with chronic pain grade score (GCPS) was evaluated. The GCF level of IL-13 was in negative correlation with chronic pain grade score at three-month follow-up; that is, the higher the GCF IL-13 values, the lower the GCPS values and vice versa (Rho = −0.333) (Table 6). There was no significant correlation between other measured GCF cytokine levels and the GCPS at three time points (Table 6).

Using Spearman’s correlation coefficient, the association of GCF levels of pro-inflammatory cytokines with jaw functional limitation subscale score was evaluated. The GCF level of IL-8 is significantly correlated with mobility limitation (Rho = 0.382) at one-month follow-up, while there was no significant correlation between measured parameters at baseline and at three-month follow-up (Table 7).

## 4. Discussion

To our knowledge, this is the first study assessing potential molecular biomarkers in the gingival crevicular fluid of TMD patients. We hypothesized that the composition of GFC might reflect local inflammation found in masticatory muscles and temporomandibular joints in TMD patients. The purpose of this study was to determine GCF levels of pro-inflammatory cytokines before and after the start of SS therapy and to explore the effect of SS on changes in the GCF cytokine levels, as well as the relationship between cytokine concentration and chronic pain intensity. To test our hypothesis, we analyzed six different cytokines that were most frequently detected in previous studies on TMDs [10,11,12]. The results showed that the GCF levels of IL-7 and IL-13 were significantly decreased at the three-month follow-up indicating that SS therapy might have a role in reducing inflammation and that the GCF could be a valuable medium for assessing molecular biomarkers. A significantly higher number of participants without pain and with low-intensity pain without disability as well as a significant decrease in jaw function limitations at the three-month follow-up confirmed the positive effect of SS on the improvement of symptoms in TMD patients. Furthermore, a correlation was found between IL-13 and chronic pain grade as well as a correlation between IL-8 and mobility limitation at one-month follow-up.

IL-7 is an immunostimulatory cytokine known for its important role in the entire lymphoid system. It is produced by lymphoid organs and nonlymphoid cells including mucosal, epithelial, skeletal muscle, and various immune cells, which further promote T and B lymphocyte development, maturation, and homeostasis [36]. Furthermore, IL-7 is linked to osteoclastogenesis and fibroblast activation, processes involved in tissue destruction in chronic inflammatory diseases like rheumatoid arthritis, ankylosing spondylitis, and inflammatory bowel disease [37,38]. Previous studies associated IL-7 with pain in patients with cancer [39] and also with myalgic encephalomyelitis, which has main symptoms similar to those of TMD patients such as fatigue, immune dysfunction, and musculoskeletal pain [40]. The number of studies investigating IL-7 concentrations in patients with TMDs is scarce. We found only two studies from the same research group that examined cytokine levels in TMD patients with jaw muscle pain. The first study reported elevated masseter muscle levels of IL-6, IL-7, IL-8, and IL-13 in patients with TMD myalgia compared to a control group [16]. IL-7, IL-13, and TNF cytokine levels increased further in response to experimental tooth-clenching, as well as jaw muscle pain and fatigue, but there were no correlations between cytokine levels and pain [16]. The second study reported that IL-7 was among nine plasma proteins that could distinguish TMD myalgia from healthy, pain-free controls with the highest discriminative power (66% higher than controls) [41]. They also reported that IL-7 was one of 12 proteins that could distinguish myofascial pain and myalgia [41]. Furthermore, IL-7 is also known as myokine, a protein synthesized and released from muscle fibers in response to physical exercise, and has been associated with tissue damage and ischemia [42]. In the context of the aforementioned studies, our results suggest that the decreased GCF levels of IL-7 resulting from SS therapy might be due to reduced masticatory muscle activity. This statement is in line with a recent study that examined the effect of SS on masticatory muscle activity in TMD patients using electromyography (EMG). The patients’ EMG values were higher than those of the control group at baseline, but there was no difference between these two groups after therapy [43]. 

IL-13 is a pleiotropic cytokine that stimulates a wide range of innate and adaptive immune cells and regulates inflammatory and immune responses [44]. It is produced by T-helper 2 (Th2) cells and plays an important role in many human diseases including systemic sclerosis, gastrointestinal inflammatory disease, inflammatory arthritis, and asthma. As we mentioned before, Jounger et al. reported elevated intramuscular levels of IL-13 in myalgia, whose values further increased during experimental tooth-clenching [16]. Furthermore, Tufvesson et al. reported higher plasma levels of IL-13 in asthma patients and controls after exercise [45] while Knudsen et al. reported that endurance exercise stimulates the production of IL-13 in muscles and thus promotes adaptations and facilitates increased muscle endurance in mice [46]. Similarly to these studies, our study result of higher values of GFC IL-13 at baseline could indicate that IL-13 might play a role in increased muscle activity in our TMD patients. We could also assume that the application of SS might lead to a decrease in muscle activity and, consequently, to a reduction in GCF IL-13 values three months after the start of treatment. 

IL-1β, IL-6, IL-8, and TNF-α are small proteins associated with inflammation secondary to arthritis or disk disorders and have an important role in the onset and progression of TMDs [11,12]. As potential biomarkers of pain in TMD, they are mainly found in synovial fluid, serum, and saliva in patients with painful disk disorders, degenerative joint diseases, and inflammatory connective tissue diseases [11,12]. In contrast, there were no significant differences in GCF concentrations of pro-inflammatory cytokines IL-1β, IL-6, IL-8, and TNF-α in our study. A possible reason for this is that our tested cohort did not include patients with degenerative temporomandibular joint diseases. Therefore, severe inflammation and consequent bone and cartilage destruction in temporomandibular joints were not present. For example, de Alcântara Camejo et al. investigated the expression of IL-6 in patients with disk displacement with and without reduction and with osteoarthrosis [47]. A higher IL-6 expression was found in a group with a more severe phenotype and in a group with osteoarthritis, suggesting that in the advanced stage of the disease, inflammation is more pronounced [47]. Similarly, Güven et al. found increased levels of TNF-α in synovial fluid in advanced stages of TMJ internal derangement [14]. 

Inflammatory pain is caused by mechanical stimulation of affected tissue’s peripheral nociceptors, which leads to hypoxia and increased secretion of various inflammatory mediators such as cytokines, chemokines, and neuropeptides, resulting in pain and hyperalgesia [48]. Previous studies reported an association between cytokine levels and pain in TMD patients [11,49]. Ulmner et al. reported a correlation between TMJ palpation pain and TNF- α and IL-1β in synovial tissue of patients with internal TMJ derangement [50], while Ernberg et al. reported an association between plasma levels of IL-7 with pain catastrophizing, and plasma levels of IL-6 with pain duration in TMD myalgia patients [41]. On the contrary, in their study, Jounger et al. did not find a correlation between cytokine levels and pain in TMD myalgia patients [16]. In the current study, we found a negative correlation between the GCF level of IL-13 and GCPS at three-month follow-up. This is consistent with the results of the recent research of Son et al. [9]. They analyzed plasma cytokines in young TMD female patients and showed that IL-13 levels were decreased in the high-disability group compared to the low-disability group, although with no significant statistical difference [9]. Our result was unexpected considering that there was a decrease in IL-13 levels at the follow-up examination after three months. A possible explanation is found in patients whose symptoms did not improve three months after the treatment and who remained in a higher grade of chronic pain. IL-13 belongs to the group of type 2 cytokines that promote angiogenesis in ischemic muscle and tissue repair [51]. A recent study by Li et al. found that attenuated IL-4/IL-13 expression is associated with angiogenesis deficit in a mouse model of diabetic peripheral arterial disease, whereas IL-4/IL-13 treatment normalizes defective regeneration [51]. Hence, the reduced levels of GCF IL-13 in the higher degree of chronic pain shown in our study might be related to the pleiotropic characteristics of IL-13 and defective regeneration during IL-13 deficiency. However, this hypothesis needs to be investigated further in future research.

Another main symptom of TMD in addition to pain is limited function of the lower jaw. Vrbanović et al. investigated the long-term effectiveness of SS compared to placebo splint (PS) in chronic TMD [52]. They found that SS was more effective in reducing spontaneous pain and functional limitations of the mandibula, with significantly higher values of pain-free mouth opening compared to patients treated with PS [52]. This is in line with our study results of significant JFLS overall score reduction after three months as well as significantly higher values of pain-free mouth opening at the end of therapy. Furthermore, we observed that the GCF level of IL-8 is positively correlated with mobility limitation, a subscale score from the JFLS questionnaire, one month after the start of the therapy. Similarly, Son et al. found a negative correlation between the plasma level of IL-8 and maximum mouth opening at three-month measurements pointing to a significant effect of IL-8 on jaw function [9]. IL-8 belongs to the CXC family of chemokines that attracts neutrophils and it is expressed in human skeletal muscle fibers in response to exercise probably to stimulate angiogenesis [53,54]. In addition, IL-8 has been associated with hypoxia, leading to an increase in IL-8 expression in skeletal muscle cells [55] and human fibroblast-like synoviocytes in patients with rheumatoid arthritis [56]. Ulmner et al. found an increased IL-8 concentration in synovial tissue in patients with TMJ disk displacement [57] and in a later study, they reported a significant correlation between TMJ synovial fluid and synovial tissue with regard to IL-8 concentration [50]. These findings suggest that IL-8 might have a role in muscle and joint tissue inflammation as a pathological mechanism that could interfere with the lower jaw’s physiological movements.

This research had rigorous exclusion criteria for the study population with a detailed clinical examination to avoid oral and systemic factors that could affect the level of inflammatory mediators. Furthermore, patients were diagnosed with TMD using a well-established and validated protocol developed for clinical and research applications. However, our study has some limitations that need to be addressed. The sample size was too small to stratify patients into diagnostic subgroups in order to examine differences in cytokine concentrations between different subgroups of TMD patients. Furthermore, in our study, gender related differences to pain were not examined since TMD more frequently affects women and our cohort consisted predominantly of females. However, this could be interesting to evaluate further in future research. In addition, the small number of male participants in our study could bias the results, so further research is needed to examine whether our results of the investigated TMD biomarkers can be applied to males to the same extent as to females. Also, one might argue that differences observed in cytokine levels might be influenced by sex hormones [58]; however, this should be minor since the cytokine levels were measured in GFC. Another limitation could be that improvements in clinical outcomes were biased by the informed examiner; however, the measurements were performed following standardized protocols and observer bias should be minimal in this regard. Also, a recent study suggested that blinding is less important than is often assumed [59]; however, replicating our study with blinding as a methodological safety measure would be valuable to perform in the future. A potential limitation of the study could be the small effect size. Although the *p* value was significant for assessments of mandibular movement, restrictions of jaw function, and the concentration of pro-inflammatory cytokines in GCF due to a relatively small effect size, one might argue that these results are not clinically significant [60]. However, these results raise the question of whether the observed differences could be of use in clinical settings. Therefore, it will be interesting to repeat this study in a larger cohort with an equal number of male and female participants.

## 5. Conclusions

This study demonstrated a positive effect of SS on clinical symptoms in TMD patients, with a significantly higher number of participants without pain and with low-intensity pain without disability as well as a decrease in jaw function limitations three months after the start of the therapy. Furthermore, results showed significantly decreased IL-7 and IL-13 GCF levels at the three-month follow-up, as well as a correlation between IL-13 and chronic pain grade and a correlation between IL-8 and mobility limitation at one-month follow-up. Our study results indicate that SS therapy might have a role in reducing inflammation and that the GCF could be a valuable source of molecular biomarkers in patients with TMD. Nevertheless, this needs to be investigated further in randomized controlled studies with larger sample sizes. 

## Figures and Tables

**Table 1:** Basic characteristics of the participants.

Gender [n (%)]	
Male	5 (14)
Female	31 (86)
Age (years) [Median (interquartile range)]	35 (28–50)
Marital status [n (%)]	
Married	16 (44)
Living as married	2 (6)
Widowed	1 (3)
Divorced	1 (3)
Never married	15 (42)
Level of education [n (%)]	
High school	12 (33)
Started, but did not graduate from college	7 (20)
College graduate	12 (33)
Professional or post-graduate level	5 (14)
Pain characteristics [Median (interquartile range)]	
Pain duration (years)	1 (0.5–3)
Current pain intensity (NRS)	4 (3–6)
Average pain intensity (NRS)	5 (4–6)

NRS—numeric rating scale.

**Table 2:** Distribution of participants in regard to opening movements, lateral movements, and protrusion at the three measurement points.

	Median (Interquartile Range)			p *	
	Baseline (T0)	One-Month Follow-Up (T1)	Three-Month Follow-Up (T2)		Effect Size ‖
Opening movements					
pain-free mouth opening	38 (35–40.5)	41 (39–45.5)	44 (41–48.5)	<0.001 ^†^	0.586
maximum unassisted	42 (39–46)	44.5 (42–48.5)	46 (43–50)	<0.001 ^†^	0.338
maximum assisted	45 (41.5–50)	45 (42.75–51)	46 (43.75–51.25)	<0.001 ^†^	0.334
Lateral and protrusive movements					
right lateral	10 (7–12)	11 (9–12)	11 (10–12)	<0.001 ^†^	0.280
left lateral	13 (11–15)	14 (12–15)	13 (11–15)	0.003 ^‡^	0.159
protrusion	6 (5–8)	7 (6–9)	8 (6–9)	0.02 ^§^	0.025

* Friedman’s test (post hoc Conover); ‖ Kendall’s W. † at the *p* < 0.05 level, there are significant differences between all three measurements. ‡ at the *p* < 0.05 level, there are significant differences between T0 vs. T1. § at the *p* < 0.05 level, there are significant differences between T0 vs. T1 and T2.

**Table 3:** Participants according to the chronic pain grade at three time points.

	n (%) Participants			p *
	Baseline (T0)	One-Month Follow-Up (T1)	Three-Month Follow-Up (T2)	
0—None	0	2 (3)	12 (33)	<0.001
I—Low-intensity pain, without disability	10 (28)	27 (75)	18 (50)	
II—High-intensity pain, without disability	13 (36)	4 (11)	1 (3)	
III—Moderately limiting	13 (36)	3 (8)	2 (6)	
IV—Severely limiting	0	0	0	

* *p*-value was calculated using the marginal homogeneity test (comparison T0 vs. T2).

**Table 4:** Differences in jaw function limitation assessment (JFLS-20).

	Median (Interquartile Range)			p *	
	Baseline (T0)	One-Month Follow-Up (T1)	Three-Month Follow-Up (T2)		Effect Size ‖
Mastication limitation	3 (2–4.75)	3.1 (1.5–4.5)	1.1 (0.4–1.9)	<0.001 ^†^	0.584
Mobility limitation	1.6 (0.7–2.5)	1.2 (0.4–3)	0.2 (0–1.4)	<0.001 ^†^	0.461
Verbal and Emotional Expression Limitation	0.7 (0–2.1)	0.5 (0–2.4)	0 (0–0.65)	<0.001 ^†^	0.341
Global	2.5 (1.5–3.5)	0.9 (0.6–2.2)	0.5 (0.1–1.4)	<0.001 ^‡^	0.595

* Friedman’s test (post hoc Conover); ‖ Kendall’s W. † at the *p* < 0.05 level, there are significant differences between T2 vs. T0, T1. ‡ at the *p* < 0.05 level, there are significant differences between all measurements.

**Table 5:** Levels of pro-inflammatory cytokines in gingival crevicular fluid (GCF) at three time points.

	Median (Interquartile Range)			p *	
	Baseline (T0)	One-Month Follow-Up (T1)	Three-Month Follow-Up (T2)		Effect Size‖
**GCF**					
IL-1ß	316.16 (148.74–731.22)	319.17 (163.12–809.34)	237.01 (108–492.61)	0.07	0.076
IL-6	9.06 (7.4–12.14)	10.06 (7.46–13.16)	9.4 (8.46–14.22)	0.74	0.009
IL-7	0.89 (0.62–1.07)	0.93 (0.7–1.23)	0.7 (0.59–0.89)	0.008	0.143
IL-8	719.89 (317.19–1022.64)	724.73 (303.27–1324.17)	480.24 (230.16–1020.44)	0.40	0.027
IL-13	2.19 (1.42–3.36)	1.9 (1.75–3.36)	1.75 (1.31–2.19)	0.003	0.175
TNF α	3.34 (2.66–4.74)	4.31 (2.57–5.77)	3.85 (2.66–4.98)	0.82	0.006

* Friedman’s test (post hoc Conover); ‖ Kendall’s W.

**Table 6:** Correlation of GCF levels of pro-inflammatory cytokines with chronic pain grade score (GCPSv2) (Spearman’s correlation coefficient).

	Spearman Correlation Coefficient Rho (*p* Value) of Chronic Pain Intensity According to GCPSv2		
	Baseline (T0)	One-Month Follow-Up (T1)	Three-Month Follow-Up (T2)
**GCF**			
IL-1ß	−0.05 (0.77)	0.107 (0.53)	0.005 (0.98)
IL-6	−0.094 (0.59)	−0.088 (0.61)	−0.069 (0.70)
IL-7	0.069 (0.69)	−0.018 (0.92)	−0.189 (0.28)
IL-8	0.075 (0.66)	0.085 (0.62)	−0.202 (0.25)
IL-13	0.072 (0.68)	−0.028 (0.87)	−0.333 (0.04)
TNF α	0.229 (0.18)	−0.120 (0.49)	−0.133 (0.45)

**Table 7:** Association of GCF levels of pro-inflammatory cytokines with jaw functional limitation subscale score at three time points (Spearman’s correlation coefficient).

	Spearman’s Correlation Coefficient Jaw Functional Limitation According to JFLS			
	Mastication Limitation	Mobility Limitation	Verbal and Emotional Expression Limitation	Total JFLS
Baseline (T0)				
IL-1ß	−0.012 (0.95)	−0.088 (0.61)	−0.072 (0.68)	−0.101 (0.56)
IL-6	−0.125 (0.47)	−0.184 (0.28)	−0.076 (0.66)	−0.164 (0.34)
IL-7	−0.054 (0.76)	−0.186 (0.28)	−0.219 (0.20)	−0.158 (0.36)
IL-8	−0.017 (0.92)	0.008 (0.96)	0.159 (0.35)	−0.009 (0.96)
IL-13	−0.013 (0.94)	−0.005 (0.98)	−0.047 (0.79)	−0.006 (0.97)
TNF α	−0.081 (0.64)	−0.051 (0.77)	−0.214 (0.21)	−0.120 (0.49)
One-month follow-up (T1)				
IL-1ß	0.199 (0.24)	0.294 (0.08)	0.165 (0.34)	0.253 (0.14)
IL-6	0.113 (0.51)	0.095 (0.58)	−0.020 (0.91)	0.080 (0.64)
IL-7	0.011 (0.95)	0.041 (0.81)	−0.058 (0.74)	0.008 (0.96)
IL-8	0.136 (0.43)	0.382 (0.02)	0.245 (0.15)	0.268 (0.11)
IL-13	−0.017 (0.92)	−0.072 (0.68)	−0.246 (0.15)	−0.040 (0.82)
TNF α	−0.043 (0.80)	0.168 (0.33)	−0.020 (0.91)	0.071 (0.68)
Three-month follow-up (T2)				
IL-1ß	0.150 (0.41)	0.153 (0.40)	0.112 (0.53)	0.126 (0.49)
IL-6	0.092 (0.61)	−0.009 (0.96)	0.101 (0.58)	0.028 (0.88)
IL-7	−0.195 (0.28)	−0.003 (0.99)	−0.176 (0.33)	−0.087 (0.63)
IL-8	−0.076 (0.67)	0.082 (0.65)	−0.042 (0.82)	0.058 (0.75)
IL-13	−0.085 (0.64)	−0.042 (0.82)	−0.145 (0.42)	−0.102 (0.57)
TNF α	−0.036 (0.84)	0.034 (0.85)	−0.053 (0.77)	−0.103 (0.57)

## Data Availability

The datasets generated and/or analyzed during the current study are not publicly available due to the fact that individual privacy could be compromised; however, they are available from the corresponding author on reasonable request.
